# Favorable Radiographic Response in a Patient With an Oligodendroglioma Treated With Azacitidine and Venetoclax for Acute Myeloid Leukemia

**DOI:** 10.7759/cureus.61540

**Published:** 2024-06-02

**Authors:** Mauricio Perez, Vanessa Barrionuevo, Cristina Arias, Joachim M Baehring

**Affiliations:** 1 Neuro-Oncology, Yale University, New Haven, USA; 2 General Practice, Universidad de Guayaquil, Guayaquil, ECU; 3 General Practice, Universidad del Azuay, Cuenca, ECU

**Keywords:** bcl2 inhibitor, hypomethylating agents, secondary leukemia, recurrent glioma, oligodendroglioma, brain neoplasm

## Abstract

The standard chemotherapy for treating oligodendrogliomas consists of a combination of procarbazine, lomustine, and vincristine (PCV). The combination of hypomethylating agents like azacitidine and BCL2 inhibitors like venetoclax has not been formally studied in the treatment of glial tumors. The combination of these two drugs is commonly used to treat acute myeloid leukemia (AML), with IDH-mutant disease being a particularly sensitive subtype. The use of azacitidine for the treatment of IDH-mutant gliomas has been reported in the literature, with mixed results that might suggest at least some benefits in a subtype of patients. It is also reported in the literature that the BCL2 gene is associated with treatment resistance and tumor recurrence in gliomas. Here, we present a patient with an oligodendroglioma who was treated with a conventional chemotherapy regimen for AML and, at the same time, had a favorable radiographic response to his brain tumor.

## Introduction

Oligodendrogliomas are rare IDH-mutant neuroepithelial tumors with a distinctive histologic appearance and molecular profile. They account for 5% of all primary brain neoplastic lesions and are more prevalent in middle-aged men [1-3]. The relative five-year survival is around 79.5%, but many factors can affect prognosis, like tumor grade, type of treatment, and individual patient’s co-morbidities [2]. Treatment options include a combination of surgery, radiation, and chemotherapy, depending on each patient's characteristics, tumor grade, and location [2]. The standard chemotherapy for oligodendrogliomas consists of a combination of procarbazine, CCNU [lomustine: 1-(2-chloroethyl)-3-cyclohexyl-1-nitrosourea], and vincristine (PCV). Although infrequent, treatment-related acute leukemias are life-threatening complications of alkylating agents. Other treatment options, like hypomethylating drugs and BCL2 inhibitors, are commonly used to treat IDH-mutant hematologic malignancies like acute myeloid leukemia (AML) but have not been formally studied in the treatment of IDH-mutant gliomas. We describe an interesting case of a patient with a relapsed grade 2 oligodendroglioma, who showed a favorable radiographic response after treatment of AML. We then discuss the possible role of hypomethylating agents and BCL2 inhibitors in patients with recurrent IDH-mutant gliomas.

This article was previously presented as a meeting poster at the 2023 SNO Society of Neuro-Oncology Annual Meeting on November 18, 2023.

## Case presentation

**Figure 1:**
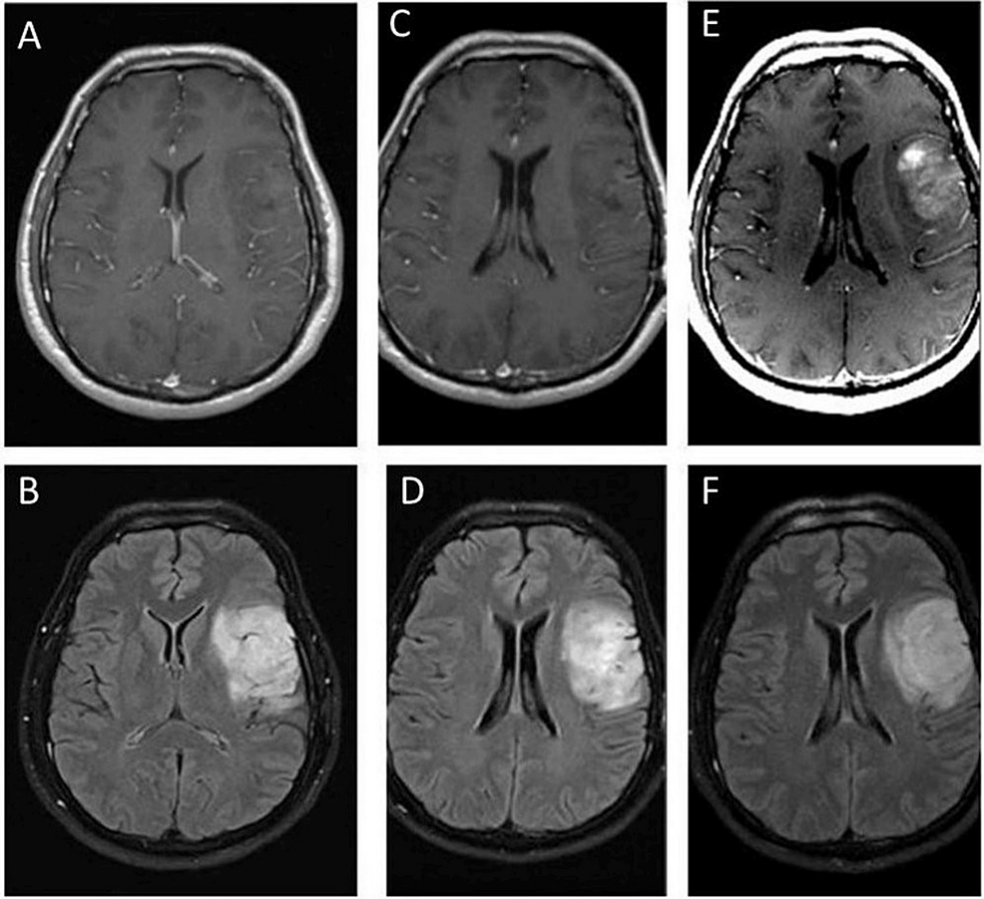
Tumor progression Axial MRI brain. T1 with contrast (top panels) and T2/FLAIR (bottom panels) A-B. Two years after biopsy of the tumor. C-D. Ten months after chemotherapy with PCV. E-F. Tumor enhancement 39 months after chemotherapy with PCV. PCV: Procarbazine, lomustine, and vincristine

**Figure 2:**
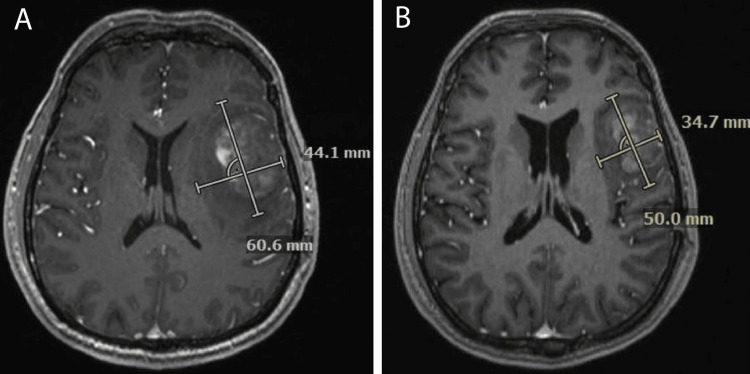
Radiographic response after three cycles of azacitidine and venetoclax Axial MRI brain with contrast. A. Tumor measurements before exposure to AZA/VEN. B. Decrease in the tumor volume of approximately 19.1% per RANO criteria. AZA/VEN: Azacitidine, venetoclax

**Figure 3:**
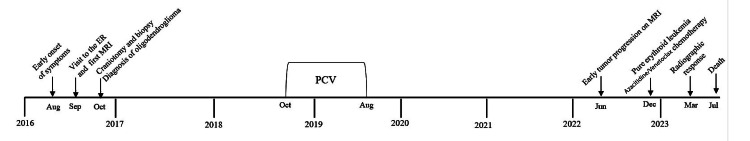
Timeline of events In August 2016, he had his first focal seizure. In September 2016, he went to the hospital after a generalized tonic-clonic seizure. The MRI of his brain revealed a left frontal tumor. In October 2016, he underwent a stereotactic biopsy, which was consistent with grade 2 oligodendroglioma. In October 2018, he had increased frequency of seizures and started chemotherapy with PCV. In June 2022, his MRI showed findings of tumor relapse but he declined further treatments. In December 2023, he was diagnosed with AML and was treated with AZA/VEN. In March 2023, a repeat MRI demonstrated a reduction in tumor volume. In July 2023, he passed away. AZA/VEN: Azacitidine, venetoclax. MRI: magnetic resonance image. PCV: procarbazine, lomustine, and vincristine.

In August 2016, a 48-year-old man presented with focal seizures consisting of speech impairment and right-hand numbness. An MRI of his brain revealed a non-enhancing left frontal mass lesion with associated vasogenic edema. Two months later, he underwent a stereotactic brain biopsy. The pathology, immunohistochemistry, and gene sequencing analyses revealed an IDH1 R132H mutant, 1p19q co-deleted, WHO grade 2 oligodendroglioma. He decided to hold off on any additional treatments after the procedure. Two years later, he had several breakthrough seizures despite good compliance with his anti-seizure medication. Although a repeat MRI of the brain was unchanged, he underwent treatment with six cycles of PCV (procarbazine 60 mg/m^2^, lomustine 110 mg/m^2^, and vincristine 1.4 mg/m^2^). After three years of clinical and radiographic stability, an MRI eventually showed signs of tumor recurrence (Figure 1), and, unfortunately, he was also diagnosed with AML shortly after. His leukemia was treated with three cycles of azacitidine and venetoclax (AZA/VEN, 75 mg/m^2^/day and 100 mg/day, respectively). Despite failure of this protocol to put his AML in remission, a repeat MRI of his brain revealed a decrease in the tumor volume of approximately 19.1%, per RANO criteria (Figure 2). Regrettably, he passed away from complications of bone marrow failure a few months later. A timeline of events is summarized in Figure 3.

## Discussion

An oligodendroglioma is a rare type of neuroepithelial brain tumor that arises from glial cells called oligodendrocytes [2]. According to the 2021 WHO classification, they can be further classified as grade 2 or 3, depending on the mitotic index, also known as the ki-67. Both must have either IDH1 or IDH2 mutations and the characteristic co-deletion of chromosome arms 1p and 19q. Oligodendrogliomas represent around 5% of all central nervous system tumors, and the average age of diagnosis is between 40 and 45 [3]. They are more common in males and more frequently diagnosed in the Caucasian population [4]. Symptoms can vary depending on their location in the brain but may include headaches, seizures, changes in personality or behavior, and focal neurological deficits. The specific treatment plan is highly individualized and depends on the tumor’s location, size, and grade. Surgery is the mainstay of treatment in most cases; however, observation might be considered for selected low-risk scenarios (i.e., patients younger than 40 years old or small tumors with no associated symptoms). According to data from the clinical trial RTOG 9802, there is both progression-free survival (PFS) and overall survival (OS) benefit for adjuvant chemotherapy and radiation, as compared to radiation alone for high-risk grade 2 glioma patients (subtotal resection or age > 40; PFS-10: 51% vs 21%; OS-10: 60% vs 40%; p=0.003) [5]. In some selected cases, radiation can be deferred due to concerns of long-term cognitive impairment, especially in the younger patient population. When treatment is tolerated, the OS for grade 2 oligodendroglioma patients is estimated to be between 10 and 20 years, and the median survival for grade 3 oligodendroglioma is around 14 years [4]. These patients often require long-term follow-up care to monitor for tumor recurrence.

Temozolomide (TMZ) and the three-drug regimen procarbazine, lomustine, and vincristine (PCV) are the main chemotherapeutic regimens for these glial tumors. Weller et al. found that PCV therapy results in long progression-free survival rates (9.1 years) and low rates of histologic progression compared to temozolomide therapy (PFS of 3.6 years) in patients with oligodendroglioma grade 2 [6]. However, up to 40% of patients develop significant toxicity. The most common grade 1 adverse effects are nausea (33%), vomiting (80%), fatigue (37%), anorexia (18%), anemia (26%), and leukopenia (36%). The most common grade 3 adverse effects include myelosuppression (63%), neutropenia (30-35%), and thrombocytopenia (up to 24%) [7,8]. There is still much debate about whether treatment with TMZ or PCV is superior to the other. However, some patients are treated with TMZ instead of PCV due to concerns of sustained hematologic toxicity and bone marrow dysfunction associated with PCV therapy.

Alkylating agents, such as nitrosureas (lomustine), carry a potential risk for the development of leukemias due to cumulative myelosuppression. Even though the development of AML or myelodysplastic syndrome (MDS) following PCV therapy is a rare and severe complication, there are several cases reported in the literature. The risk of developing MDS or AML is related to the type of chemotherapy used, the dose, treatment duration, and patient comorbidities [9]. 

Our patient received three cycles of AZA/VEN. Despite the progression of his leukemia with this treatment, there was radiographic evidence that the oligodendroglioma decreased in size while he was on treatment with azacitidine/venetoclax. Azatidine reduces cell growth by reducing CpG hypermethylation and increases glial fibrillary acid protein expression. At the same time, it enhances the therapeutic effect of temozolomide when used in combination [10,11]. In the case series by Federici et al., one patient with an IDH-mutant oligodendroglioma out of five had a sustained stabilization with 5-azacitidine used for 22 cycles [12].

Somatic IDH mutations are present in solid and hematologic malignancies (myelodysplastic syndrome and acute myelogenous leukemia). However, IDH1 is more common in low-grade gliomas, while IDH2 mutations are more prevalent in hematologic malignancies. Chan et al. discovered that patients with IDH1/2 mutation are more likely to respond to BCL2 inhibition by venetoclax [13]. IDH1-mutant gliomas manifest the cytosine-phosphate-guanine (CpG) island methylator phenotype (G-CIMP+) [14]. Noushmehr et al. found that G-CIMP is associated with a younger time at diagnosis (median age 36; p<0.0001) and secondary or recurrent gliomas [15].

Currently, there are two FDA-approved drugs to treat IDH-mutated myelodysplastic syndromes: Ivosidenib (mutIDH1) and Enasidenib (mutIDH2). However, their application remains limited due to acquired resistance caused by secondary mutations [16]. The recently published INDIGO trial (NCT04164901) is a phase 3 study that analyzed the use of vorasidenib in patients with grade 2 IDH-mutant residual or recurrent gliomas. The data revealed improvement in progression-free survival (HR 0.26; 95% CI (0.15-0.43); p=0.000000019) with minimum toxic effects [17].

The application and effectiveness of hypomethylating agents and BCL-2 inhibitors in solid tumor malignancies remain in study, as there is limited experience. The radiographic response of our patient's oligodendroglioma to AZA/VEN is encouraging; nonetheless, further investigations are needed.

## Conclusions

The treatment options for recurring low-grade gliomas are limited and may involve surgical debulking of the mass, additional chemotherapy, or brain irradiation. A recent trial testing vorasidenib, an IDH-mutant inhibitor for low-grade glioma, seems to be a promising alternative to patients with residual or recurrent disease and is pending FDA approval. 

Interestingly, treating our patient's leukemia with azacitidine and venetoclax induced a partial radiographic response to his brain tumor. The combination of hypomethylating agents and BCL2 inhibitors is a standard treatment for acute myeloid leukemias, which can harbor an IDH-2 mutation. Further studies are needed for IDH-mutant gliomas, for which treatment options are still limited. Based on these findings, more information from additional case reports or case series is needed to study the safety and efficacy of this chemotherapy protocol in clinical trials and other prospective studies. 
